# Video‐based interventions promoting social behavioural skills for autistic children and young people: An evidence and gap map

**DOI:** 10.1002/cl2.1405

**Published:** 2024-05-03

**Authors:** Karen McConnell, Ciara Keenan, Catherine Storey, Allen Thurston

**Affiliations:** ^1^ School of Nursing and Midwifery Queen's University Belfast Belfast UK; ^2^ Research and Evidence, National Children's Bureau London UK; ^3^ School of Educational Sciences Bangor University Bangor UK; ^4^ School of Social Sciences, Education and Social Work Queen's University Belfast Belfast UK

## Abstract

**Background:**

Video‐based interventions (VBIs) are an approach that can be used to promote social behavioural skills for autistic children and young people. Despite an abundance of literature in this area, previous evidence syntheses are limited by their exclusive search strategies and eligibility criteria. Therefore, there is a lack of comprehensive evidence syntheses to provide insight on whether these interventions work, for whom, and in what circumstances. Evidence and Gap Maps (EGMs) are used to collate vast literature on a broad topic area such as this, highlighting areas for synthesis, and identifying gaps for future research.

**Objectives:**

To identify, map and synthesise existing primary research on VBIs promoting social behavioural skills for autistic children and young people, creating a live, searchable and publicly available EGM.

**Search Methods:**

Searches were conducted in electronic databases (*n* = 8), web search engines, and other repositories including published papers and grey literature. The search strategy was developed around two concepts including (1) terms related to autism, and (2) terms related to VBIs. Searches were conducted in May 2021.

**Selection Criteria:**

All primary studies evaluating the effectiveness of VBIs in promoting social behaviours for autistic children and young people aged 3–18 were included in the EGM.

**Data Collection and Analysis:**

Search results were imported into Eppi‐Reviewer where duplicates of identical studies were removed. Titles and abstracts were then screened by two independent reviewers. Potentially eligible full texts were located and also screened by two reviewers. Data were then extracted on study design, participant characteristics, type of intervention, type of outcome, and country of study, by one of three reviewers. EPPI‐Mapper was used to create the interactive EGM.

**Main Results:**

The current EGM contains 438 studies reporting on 394 single subject research designs, 25 randomised controlled trials, 15 non‐randomised group designs, and 8 pretest–posttest designs. Included studies evaluated VBIs in all male (*n* = 238), mixed gender (*n* = 172) or all female (*n* = 17) samples. VBIs employed included video modelling (*n* = 273), video self‐modelling (*n* = 82), point‐of‐view modelling (*n* = 61), video prompting (*n* = 57), video feedback (*n* = 12) and computer‐based video instruction (*n* = 4). The most frequently used models were adults (*n* = 191) and peers (*n* = 135). In relation to social outcomes, almost half evaluated social engagement (*n* = 199) with limited studies looking at safety (*n* = 9) and community (*n* = 7) skills.

**Authors' Conclusions:**

This EGM provides a valuable resource for policy‐makers, practitioners, researchers, funders and members of the public to access evidence on VBIs promoting social behavioural skills in autistic children and young people. The map has identified areas of sufficient research where evidence can undergo synthesis. In addition, important gaps in the evidence were highlighted and suggest further research is warranted in all female samples and less frequently evaluated types of VBIs and social outcomes. Evidence included in this EGM will be further explored via systematic review and meta‐analysis on control group designs.

## 1 PLAIN LANGUAGE SUMMARY

This evidence and gap map (EGM) of video‐based interventions (VBIs) for autistic children and young people is unevenly distributed with respect to geography, gender and type of intervention; with most studies focussed on male participants, younger children, and conducted in high income countries.

### 1.1 The EGM in brief

There is a considerable body of evidence related to VBIs for autistic children and young people, but it is unevenly distributed by geography, gender, and type of intervention and outcome.

### 1.2 What is this EGM about?

Autism is a lifelong condition affecting around 1 in 100 children and young people worldwide. Autistic children and young people experience challenges with social interaction and communication that can result in problem behaviours.

Policy‐makers and practitioners need to know what VBI works, for whom, and in what circumstances, to improve and plan services for autistic children and young people. This EGM summarises the available evidence from research evaluating the effectiveness of VBIs promoting social behavioural skills in autistic children and young people.

### 1.3 What is the aim of this EGM?

The aim of this EGM is to show all the available evidence from primary research studies presenting data on VBIs promoting social behavioural skills in autistic children and young people aged 3–18 years.

### 1.4 What studies are included?

The current map includes 438 studies: 394 single‐subject research designs, 25 randomised controlled trials (RCTs), 15 non‐randomised group designs, and 8 pretest–posttest designs. The majority of studies were carried out in the United States of America. The studies were categorised according to the type of intervention used and the type of social outcome measured.

### 1.5 What are the main findings of this gap map?

The majority of studies come from high‐income countries (*n* = 409) with a very small number from low and middle‐income countries (*n* = 29).

The studies are unevenly distributed across intervention types with video modelling the most common type of VBI: 273 of the 438 studies evaluate video modelling. In comparison, only four studies use computer‐based video instruction.

The most frequent social outcomes are social engagement (*n* = 199) and activities of daily living (*n* = 84) with very few studies considering safety (*n* = 9) and community (*n* = 7) skills.

More than half of studies look at VBIs only in males and most include children of pre‐school/primary school age (*n* = 371).

### 1.6 What do the findings of the map mean?

The current EGM provides a summary of the available research evidence on the effectiveness of VBIs promoting social behavioural skills for autistic children and young people, to researchers, policy‐makers and practitioners. Whilst the evidence base is relatively large, there is a need for more research with autistic females, using cartoon/animated models, considering safety and community skills, and carried out in low and middle‐income countries. In addition, this map highlights important areas for synthesis and analysis to determine which VBIs are effective, for whom, and why.

### 1.7 How up‐to‐date is this EGM?

The authors searched for studies published up to May 2021.

## 2 BACKGROUND

### 2.1 Introduction

#### 2.1.1 The problem, condition or issue

Autism is a lifelong neurodevelopmental condition that is characterised by differences in social interaction and communication, and repetitive and restrictive behaviours (World Health Organisation, 2022). The prevalence of autism is rising, due in part to increased public awareness, improved assessment methods and earlier identification (Fombonne, 2005; Zeidan et al., 2022). Currently, it is estimated that around 1 in 100 children and young people are diagnosed as being autistic (Zeidan et al., 2022). Whilst gender based differences in autistic traits have been evidenced (Ratto et al., 2018), males remain three times more likely to be diagnosed with autism than females (Loomes et al., 2017).

Due to differences in social interaction and communication, autistic children and young people face difficulty in expressing their wants and needs which may subsequently result in problem behaviours such as aggression and self‐injury as an alternative communication strategy (Carroll et al., 2014; Chiang, 2008; Murphy et al., 2009). Sensory seeking behaviours, often experienced by autistic people can be misinterpreted as problem behaviours despite them being reported as useful behaviours by autistic persons (Kapp et al., 2019). In addition, autistic children and young people tend to have a lack of safety awareness and are subsequently at increased risk of injury than their non‐autistic peers (Jain et al., 2014; Lee et al., 2008).

VBI is an instructional method whereby an individual will watch a video of a model performing a skill in its entirety and then attempt to complete the skill in the same way (LeBlanc et al., 2003). Research has shown that VBI can be particularly useful for autistic individuals because videos can be broken into clips which promotes skill chaining and reduces the engagement time necessary with the video (Buggey, 2007). VBI also provides support to autistic children and young people who experience differences in complex imitation skills which are necessary for observational learning to occur (Bandura, 1969). In addition, VBI combines instruction with an already preferred activity for many individuals. Research suggests that using technology and watching videos are highly reinforcing for many autistic children and young people (Anderson et al., 2018; Charlop‐Christy & Daneshvar, 2003). Instruction combined with reinforcement may increase a child's motivation to learn and perform a new skill (Hendricks & Wehman, 2009).

#### 2.1.2 Why it is important to develop the EGM

Whilst a number of reviews have been undertaken to synthesise research on VBIs promoting social behavioural skills for autistic children and young people (Bellini et al., 2007; Mason et al., 2013, 2016; Wang et al., 2011), they have not developed an advanced search strategy that optimises retrieval of all relevant research. In addition, several previous reviews focused on one type of VBI (Bellini et al., 2007; Mason et al., 2013, 2016), were not autism‐specific (Mason, 2012) or excluded papers not published in peer‐reviewed journals (Mason, 2012). The advanced search strategy developed by the lead reviewer (CK), identified a large volume of relevant research studies (i.e., >80 studies) on this broad topic, thus warranting summary in an EGM (Campbell et al., 2023). This map forms part of a larger programme of work and a subsequent systematic review and meta‐analysis is currently being conducted by the review team to answer quantitative research questions on the effectiveness of VBIs promoting social behavioural skills in autistic children and young people (Keenan et al., 2021).

The EGM is live, searchable and publicly available, providing a visual presentation of VBI research for promoting social behavioural skills in autistic children and young people. It identifies gaps in the evidence base and highlights areas of sufficient research for evidence synthesis. This enables (1) funders to quickly identify already saturated research areas, meaning resources can be directed to areas requiring further research, (2) policy‐makers and practitioners to inform policy and practice using existing evidence, (3) researchers to minimise waste and duplication of research effort, and (4) members of the public to advocate for services using relevant information that is available in a user‐friendly format.

## 3 OBJECTIVES

This EGM aimed to identify, synthesise and map existing published and unpublished evidence (primary studies) on VBIs promoting social behavioural skills for autistic children and young people. The specific social behavioural skills of interest included:

- Social engagement
- Imaginative play
- Safety skills
- Academic performance
- Community skills
- Vocational skills
- Emotional regulation
- Activities of daily living

The EGM is part of a wider programme of work (Keenan et al., 2021) including a systematic review and meta‐analysis aiming to evaluate the effectiveness of VBIs promoting social behavioural skills in autistic children and young people.

## 4 METHODS

### 4.1 EGM: Definition and purpose

EGMs collate the available evidence on a broad topic into a visual and interactive tool that is publicly available. They clearly identify areas with adequate evidence for review and gaps requiring new evidence (White et al., 2020). Due to their user‐friendly format, EGMs provide a valuable resource for informing policy, practice and research (Snilstveit et al., 2016).

### 4.2 Framework development and scope

The standard EGM framework was used as a matrix and guided eligibility criteria for the map. The columns contain information on the social outcomes such as social engagement, imaginative play, safety skills. The rows contain information pertaining to the type of VBI such as video modelling, video self‐modelling, point‐of‐view modelling. The following filters were added to the map to enable users to refine their search for studies: study design, age of participants, gender of participants, type of model, intervention setting, sample size, country. The framework and filters were originally developed using knowledge and evidence gained via completion of a previous systematic review and meta‐analysis (Keenan et al., 2016). This framework was then reviewed against stakeholder responses to an online survey described in Section 4.3. Based on these responses, no gaps in the framework were identified, and thus the framework remained unchanged. Input from key stakeholders corroborated the relevance and appropriateness of the framework.

### 4.3 Stakeholder engagement

Stakeholder engagement is recognised as an important process in evidence synthesis to shape the review, reduce bias and integrate review findings (Haddaway et al., 2017). It is preferable to engage with stakeholders during all stages of the review process however stakeholders were not involved prior to publication of the protocol (Keenan et al., 2021). The review team recognised the value of stakeholder engagement and thus commenced the process while the review was underway. An online survey was developed by the research team to ascertain stakeholder perspectives for the current EGM and systematic review. Survey questions asked about (1) key variables to be extracted, (2) important analyses not already planned for the systematic review, (3) dissemination preferences, (4) accessibility issues, and (5) other outcomes of interest for subsequent reviews. A participant information video detailing the aims of the survey, what it involves, its voluntary nature, confidentiality information and researcher contact details was disseminated to autistic persons, their carers and families, relevant professionals and organisations, via social media. Those who were interested in completing the survey were asked to click on a link taking them to the eligibility screening page and subsequent online consent form if they were eligible to take part. Once consent had been obtained, participants were directed to the survey questions. As stakeholder engagement is not research, the authors do not have permissions to share results of the survey. However, the results were used to shape data extraction, data analysis, write‐up and dissemination of the overall programme of work outlined in the published protocol.

### 4.4 Dimensions

The above EGM framework informed the inclusion and exclusion criteria. The type of VBI (e.g., video modelling, video self‐modelling) and social outcomes (e.g., social engagement, imaginative play) were chosen as the key dimensions.

#### 4.4.1 Types of study design

The EGM included all primary studies, both published and unpublished, that presented data on the effectiveness of VBIs promoting social behavioural skills for autistic children and young people. Study designs included were RCTs, quasi‐RCTs, pretest–posttest designs, posttest only (non‐equivalent groups) and single subject research designs (SSRDs).

#### 4.4.2 Types of intervention/problem

Interventions included in this EGM are the VBI types listed in Table 1, delivered to autistic individuals aged 3–18 years with a professional diagnosis and with an explicit objective of promoting social behavioural skills.

**Table 1:** Types of video‐based interventions included in the EGM.

Type of video modelling	Description	Example	Model
Video modelling	Learner watches a video of other people/another person engaging in a desired task or skill from beginning to end. When the video ends, the child imitates the behaviour.	Learner watches a video of a person completing all of the steps necessary during tooth brushing.	Adult Sibling Peer Avatar Cartoon animations
Video self‐modelling	Learner watches a video of themselves engaging in a desired task or skill (created using video editing software) from beginning to end. When the video ends, the child imitates the skill in real time.	1. Learner watches a video of themselves reading a paragraph from a textbook, without any pauses. 2. Learner watches a video of themselves making a cheese toastie from beginning to end, without any pauses or disruptions.	Learner (Self)
Point‐of‐view modelling	Video is filmed from the perspective of what the learner will see when they are performing the target behaviour. Recording typically takes place from the eye level of the model, thus the learner will only see the hands/arms of the model.	1. Learner watches a video of a person tying their shoelaces. The learner can only see the hands/arms of the model. 2. Learner watches a video of their route to work. Learner views the exact route to take to work from their front door to their office door. Learner only sees the street ahead with one shot displaying the models' hand on the door of their office.	Adult Sibling Peer Avatar Cartoon animation Self
Video prompting	The video serves as a cue for the individual steps of a task. Records someone going through the routine of brushing their teeth and breaks the video down into steps that the learner watches while completing the task. Video prompting breaks the task down into steps whereas video modelling presents the task to the learner as a whole.	1. Video prompting procedure is used to teach a learner with ASD to do their own laundry. Learner watches step 1 of the video, which shows a model separating clothes into colours/whites. Learner completes this step in real time. Learner then watches step 2, place whites in drum of machine. Learner completes this step, etc… Learner may see the model in their entirety or only the part of their body completing the task.	Adult Sibling Peer Avatar Cartoon Animation Self
Video feedback (VFB)	An instructor reviews previously filmed footage of the learner engaging in a target behaviour, with the learner present. Instructor provides feedback (or reinforcement for appropriate behaviours) and teaches the learner to discriminate between correct and incorrect responses. N.B: There are many variations of VFB, but this will always differ from video self‐modelling because feedback is provided. Feedback is not provided when a learner is played a video modelling recording.	Nine‐year‐old Adam is filmed during a group science project in a typical classroom. Prior to the next science lesson, a classroom assistant sits with Adam and reviews the video. She reinforces his conversation initiations with his peers and addresses his interruptions. They strategise that during the upcoming science lesson, he will replace his interruptions with ‘excuse me’.	Model is generally the learner (self) but this can also be an adult, peer, sibling etc. Person providing feedback is generally an adult/parent/guardian.
Computer‐based video instruction (CBVI)	CBVI provides simulated instruction within realistic, interactive learning environments through incorporation of video models and captions. This form of simulation offers multiple teaching examples that replicate the varied environments in which the skills will be used. CBVI is often presented in a very game like format.	Fourteen‐year‐old Clare's teachers decide to use CBVI to prepare Clare for independent shopping in the community. Every day, during Clare's citizenship class, her teachers show her a video on the ipad which simulates a supermarket. Clare has to put virtual items into a virtual basket and when the model in the video gets to the till and the cashier asks for £7.50, Clare has to count this out from her virtual wallet.	Adult Peer Avatar Cartoon Animation N.B: CBVI programmes are usually quite standardised so it would be unlikely that the learner would know the model, unless the programme was developed specifically for this learner.

Any studies which used video technology to collect and observe data were not included, such as an RCT of a joint attention intervention in autistic children and young people where a video was used to record teacher–child–mother interactions for outcome data. As it had no other part to play in the intervention, it was excluded (Kaale et al., 2012).

Similarly, in a Quasi RCT by Trimmer et al. (2017), 25 autistic individuals were compared to 25 matched controls on their emotional responses to a distressing video scene. As the video was not being used to teach a new social skill, but instead was being used as a tool to elicit a response, this study was also excluded.

#### 4.4.3 Types of participants

Participants were aged between 3 and 18 years old and diagnosed as autistic by a professional.

As this EGM encompassed all published studies worldwide, an inclusion strategy of age 3–18 years supported coverage for those studies which were carried out in an educational institution and were inclusive of the first stage of schooling, that is, preschool stages in the UK, until the final years of education and transition into vocational skills.

Countries such as Israel, America and Belgium have a higher school‐leaving age of 18 compared with the UK, Australia and Italy who have a school‐leaving age of 16.

Although the EGM was not limited to those interventions carried out in an educational institution, we chose the school‐age population to encapsulate this population of children and young people.

Only participants diagnosed as autistic by a professional and as defined by the DSM‐5 were included. We also included participants with comorbid diagnoses in our initial searches to reduce the risk of overlooking any relevant sources.

#### 4.4.4 Types of outcome measures

This EGM sought to map evidence on social behavioural skills in autistic children and young people. A description of the following behaviours of interest is available in Table 2:

- Social engagement with peers
- Social understanding
- Safety skills
- Imaginative play
- Academic performance
- Community skills
- Vocational skills
- Emotional regulation

**Table 2:** Types of outcomes.

Outcome	Description	Research examples
Social engagement	Difficulties instigating social interactions or interacting with others. Autistic individuals may interact with others in a manner deemed ‘odd’ or socially unacceptable. They may choose to isolate themselves from others due to difficulties instigating social interactions or maintaining appropriate social interaction. Typically developing people may avoid interacting with autistic people due to conversation which may only be of interest to the autistic person. Here we are looking for any VBIs that target this deficit by teaching the autistic person to initiate conversation, eye contact, use more socially acceptable conversation and improve their listening/conversational skills.	1. Increasing eye contact in autistic pre‐schoolers. 2. Promoting appropriate greetings for autistic high school students. 3. Improving turn‐taking in a special education classroom. 4. Increasing question‐asking of autistic school‐aged children. 5. Teaching autistic children to initiate playground games. 6. Improving ‘dinner table’ etiquette of autistic adults.
Imaginative play	Autistic children often have difficulty with role‐play, such as acting out fictional characters from books, tv‐shows, movies. In addition, they often display issues using toys such as dolls houses to act out fictional scenarios or even simulate scenarios relevant to their own lives. This type of play is important for early brain development. Autistic children tend to prefer restrictive and repetitive play such as lining objects, spinning wheels on toys, sorting. Here, we are looking for any VBIs which focus on appropriate play skills such as using dolls, props, dress up to simulate real‐life environments, and research that uses VBI in drama school/theatre settings for adult participants.	1. Increasing appropriate play with classroom toys. 2. Teaching autistic children to play dress‐up. 3. Teaching autistic children to character match a doll with their favourite superhero. 4. Engaging autistic adults in theatre productions.
Safety skills	Autistic individuals are amongst the most vulnerable individuals in our society. Safety skills that appear to develop quite naturally for typically developingindividuals, often need to be explicitly taught to autistic persons. These include fire‐safety, road safety, water safety, lure from strangers, elopement (bolting away from an adult). We will look for any intervention that uses VBI to target skills that will keep an individual safe. Sometimes, these may be referred to as ‘life skills’ but do not cover vocational or daily living skills.	1. Teaching water safety skills. 2. Increasing successful transitions during fire alarm drills. 3. Discriminating between requests from strangers and known adults. 4. Increasing hand‐holding compliance while crossing the road.
Academic performance	There are two categories to think of when identifying research that focuses on academic performance. The first, is if the intervention focuses on improving the learning environment for autistic students (changing how we teach by using targeted academic interventions). The second, is if the intervention focuses on reducing disassociation with the education system. Research that falls into either of these categories will have an overarching aim to improve overall academic performance. ‘On‐task’ behaviour is a phrase we use in autism research to simply suggest engagement. All VBIs that focus on increasing classroom ‘on‐task’ behaviour should be categorised as having an academic performance outcome.	Category 1: 1. Increasing phonemic identification of autistic children using video modelling. 2. Improving early numeracy skills of autistic children using video modelling. Category 2: 1. Increasing task engagement for autistic high school students. 2. Increasing successful transitions from recess to lessons. 3. Using a video‐based communication programme to encourage autistic students to ask for help during lessons.
Community skills	Being out in the community can pose a number of challenges for autistic individuals, including but not limited to sensory overload and environmental uncertainty. Often, autistic individuals require targeted practice at activities such as grocery shopping, attending the cinema, going to the library, using a bank machine, paying for parking etc. Point‐of‐view modelling, video self‐modelling, video modelling and computer‐based instruction can be very useful in facilitating this practice. Where there is crossover with what is a community skill and a vocational skill, we use the setting to make the final determination on what the primary outcome is.	1. Increasing independent shopping skills for autistic adults. 2. Teaching an autistic adult to lodge a cheque at the bank. 3. Increasing independence with using an ATM. 4. Returning or exchanging an item of clothing in a clothes shop.
Vocational skills	Teaching vocational skills to autistic individuals can open up a wealth of opportunities relating to employment and independent living skills. Vocational skills relate to anything which an autistic individual could do to earn a living. Including (but not limited to) teaching cashier skills, teaching book sorting skills (working at the library) rolling cutlery (café/restaurant employment), sweeping floors and cleaning (useful for hair dressing employment) cookery skills (employment in kitchens) order‐taking (waiter). Here, we are looking for any VBIs that are going to target future employment skills.	1. Teaching accurate order taking to an autistic adult. 2. Teaching autistic individuals basic porter skills. 3. Increasing accuracy with floor sweeping to autistic adolescents. 4. Teaching autistic adults to operate a till.
Emotional regulation	Many autistic individuals require explicitly taught ways to express emotions such as anger, stress and anxiety in socially appropriate ways. Often, autistic individuals can engage in aggression, property destruction, excessive crying, screaming and self‐injurious behaviour in an attempt to communicate their anger, stress or anxiety. VBI can be used to model appropriate ways of expressing and managing these emotions. Here, we are looking for any VBIs that essentially model replacement behaviours for emotional outbursts/aggressive behaviours. These will typically be functional communication skills such as asking for a break or asking for help rather than engaging in an emotional outburst.	1. Using video modelling to teach autistic children the correct way to ask for a break during numeracy lessons to decrease challenging behaviour. 2. Reducing classroom property destruction using video self‐modelling, where a student is shown a video of themselves walking to the sensory room (safe space) (Replacement behaviour). 3. Increasing successful transitions using peer modelling.
Activities of daily living	Activities of daily living include showering, using the toilet, hand washing, using/disposing of sanitary products, doing laundry,^a^ cooking,^a^ changing bedsheets,^a^ tooth brushing, getting dressed, hair brushing, washing dishes.^a^ These behaviours are typically the very basic things we do every day but these are not automatic to autistic individuals. VBIs of this nature will generally break this skill down into its component parts and display a model completing all of the steps slowly and clearly (if video modelling) or just play one of the component steps at a time (if Video Promping).	1. Using video modelling to teach hand washing steps to autistic preschool children. 2. Teaching autistic women to independently change their sanitary towels. 3. Using video modelling to teach an autistic boy to prepare an after‐school snack.

^a^ These activities could also be categorised as vocational tasks.

#### 4.4.5 Other eligibility criteria

##### Types of settings

Settings were not restricted in any form and included community based settings, vocational settings, educational institutions, after school facilities, summer schemes, treatment centres, clinical settings and the individual's home.

#### 4.4.6 Additional dimensions

Additional dimensions were added to the EGM including study design, age and gender of participants, type of model, intervention setting, sample size and country.

### 4.5 Search methods and sources

Literature retrieval methods for this EGM followed high‐quality standards and all searches were conducted and reported according to Campbell Collaboration guidelines (White et al., 2020). A search strategy was developed according to Kugley et al. (2017) and piloted by information retrieval specialist author (CK) in collaboration with the rest of the review team. It was expected that literature would be widely scattered due to the many differences in terminology. The lack of similarity used within bibliographic databases for indexing keywords was also acknowledged (Dixon‐Woods et al., 2006). Therefore, the ‘pearl harvesting’ method (Sandieson, 2006; Sandieson et al., 2010) that has been used successfully in a previous systematic review (Waddington et al., 2014) was adopted to find all relevant keywords to ensure the search was as inclusive as possible. The search strategy was built around the population and intervention of interest, and the pilot Medline (Ovid) search strategy is detailed in Table 3.

**Table 3:** Medline (Ovid) search strategy.

1	exp Autistic Disorder/	20,995
2	exp Asperger Syndrome/	1770
3	exp Autism Spectrum Disorder/	32,380
4	(autis* or ‘pervasive developmental disorder*’ or ‘pervasive developmental delay*’ or ‘pervasive developmental disabilit*’ or ‘global developmental delay*’ or asperger* or ASD or HFA or HFASD or ‘HF‐ASD’ or SCD or PDD or Rett* or ‘childhood disintegrative disorder*’ OR ‘triad of impairment*’ or ‘Fragile X’ or PDDNOS or ‘PDD‐NOS’ or ‘PDD/NOS’ or savant or ‘reactive attachment disorder*’ or ‘AS/HFA’ or Kanner* or aspies or ‘childhood schizophrenia’ or ‘atypical personality development*’).ti,ab,kw.	90,650
5	or/1–4	93,407
6	(video* adj3 (intervention* or feedback or prompt* or model*)).af.	6956
7	(model* adj3 (self or peer* or ‘in‐vivo’ or ‘in vivo’)).af.	54,168
8	(‘Point‐of‐view’ or ‘Point of view’).af.	44,323
9	or/6–8	105,203
10	5 and 9	568

#### 4.5.1 Electronic searches

The following electronic databases were searched on 6‐13 May 2021:

- Medline ALL (Ovid)
- PsycINFO® (Ovid)
- Web of Science (Core Collection)
- Education Resources Information Centre (ERIC, EBSCO host)
- International Bibliography of the Social Sciences (IBSS, ProQuest host)
- SCOPUS (ELSEVIER)
- British Education Index (BEI, EBSCO host)
- The Cochrane Central Register of Controlled Trials (CENTRAL)

#### 4.5.2 Other sources

Grey and supplementary searches were carried out in the following sources:

- Google Scholar's top 1000 results retrieved using the following search terms: (autis*|asperger*|‘pervasive developmental*’|‘triad of impairment*’|‘Fragile X’)(video*) (model*| intervention*| feedback| prompt*|self|peer*|‘point of view’)
- Journal of Applied Behaviour Analysis (JABA) was hand‐searched as this is the flagship journal for behaviour analytic research
- ProQuest Dissertation and Theses (global)
- Government repositories including GOV.uk for United Kingdom and the gao.gov for the US government accountability office
- Reference lists of included studies

#### 4.5.3 Search limits

Only studies available in the English language were included in the map due to the limited language skills of the review team. There were no date limits added to the searches.

### 4.6 Analysis and presentation

The review team exported coded data from EPPI‐Reviewer 4.0 (Thomas et al., 2010) to a JSON file. This file was then imported into EPPI‐Mapper software to create a live, accessible and interactive map as an HTML file.

### 4.7 Data collection and analysis

#### 4.7.1 Screening and study selection

Results from searches were imported to EPPI‐Reviewer 4.0, a web‐based application for managing and analysing data in research synthesis (Thomas et al., 2010). Duplicate results were then removed in EPPI‐Reviewer 4.0 and all reviewers were trained in using the online software. Prior to screening, four independent reviewers (CK, CS, KMC, AT) were assigned the same batch of 100 studies. Reviewers then screened the batch of 100 studies independently and met as a team to discuss discrepancies in order to enhance consistency between raters. The lead reviewer (CK) screened all abstracts while the trained reviewers (CS, KMC, AT) were assigned batches of 500 abstracts at a time until all abstracts had been reviewed twice. Reviewers independently made decisions to either include, query or exclude an abstract and all decisions were checked for consensus and disagreements. Conflict resolution meetings were held regularly throughout the screening process to critique the clarity of inclusion and exclusion criteria. Those studies in which both reviewers independently agreed to include moved forward for full‐text screening, those studies which both reviewers independently agreed to exclude were removed from the library, and for those studies in which reviewers queried or disagreed, consensus was reached through discussion or via the involvement of a third reviewer.

After title and abstract screening, the remaining studies were carried through to full‐text screening. These studies were located and downloaded for full‐text review. The PDFs for these studies were saved to Eppi‐Reviewer 4.0 and stored under a unique study ID.

The lead reviewer (CK) and three trained reviewers (CS, KMC, AT) then used the eligibility criteria outlined above to independently decide whether the full‐text study should be included, queried, or excluded. Interrater agreement was calculated using the Statistical Package for the Social Sciences (SPSS; IBM Corp. Released 2022. IBM SPSS Statistics for Windows, Version 29.0.: IBM Corp). The Fleiss' Kappa statistic (Fleiss, 1971) was used to measure reliability across multiple raters at abstract screening; this approach assessed the degree of agreement across all studies screened by title and abstract. Due to the inability to retrieve comparison data for full text screening from Eppi‐Reviewer, the authors were unable to calculate agreement between raters in the full text screening stage.

#### 4.7.2 Data extraction and management

Data extraction forms were designed by the authors using Eppi‐Reviewer 4.0. The form was piloted by three reviewers (CK, CS, KMC) extracting data from the same eight studies. The form was subsequently refined to ensure the relevance of data and ease of extraction. Once the data extraction form had been piloted and agreed upon by the review team, one author extracted the following data for the map:

- Type of VBI: video modelling, video self‐modelling, point‐of‐view modelling, video prompting, video feedback, computer‐based video instruction
- Type of social outcome: social engagement, imaginative play, safety skills, academic performance, community skills, vocational skills, emotional regulation, activities of daily living
- Type of model: peer, self, cartoon/animated, adult, not specified
- Intervention setting: pre‐school, school, research setting, home, clinical, after school, vocational/work placement, community, holiday/summer scheme, not specified
- Study design: RCT, Non‐randomised group design, pretest‐posttest design, SSRD
- Age of participants: 3–6 years, 7–11 years, 12–15 years, 16–18 years, not specified
- Gender of participants: all male, all female, mixed gender, not reported
- Sample size: less than 3, 3–7, 8–12, 13–17, 18 or more
- Country of study: as per EPPI‐Reviewer 4.0 country list

Individual studies could only be assigned one type of study design, sample size and gender, but could be assigned more than one code in each of the remaining categories. Therefore, a single study may appear on the map in multiple locations, making the evidence base seem larger than it is.

Any studies identified as ineligible during data extraction were listed as ‘excluded’ and the reason for exclusion was recorded. Quality and consistency of extracted data were ensured via regular meetings with the review team. During this stage, weekly meetings were convened to discuss data extraction and day‐to‐day contact was maintained via email to address queries and share decisions.

#### 4.7.3 Quality appraisal

Quality appraisal was not carried out as systematic reviews were excluded and appraisal of primary studies is not considered a mandatory component in EGMs. Systematic reviews were not included due to the comprehensive searches that were carried out to identify all relevant primary studies in the English language. Primary studies will undergo quality assessment during any subsequent systematic reviews that are carried out using the map.

## 5 RESULTS

### 5.1 Description of studies

#### 5.1.1 Results of the search

**Figure 1:**
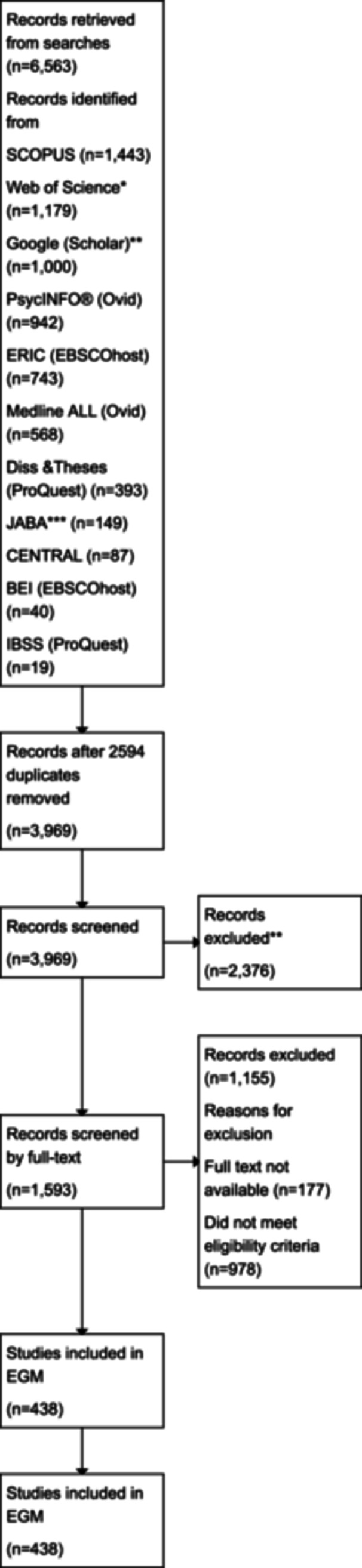
The PRISMA flow diagram details the databases searched, the number of abstracts screened, the full texts retrieved and the total number of studies included in the EGM. *Web of Science (Core Collection. Accessed through Queen's University Belfast subscription: 1970–present). Sources include Science Citation Index Expanded (SCI‐EXPANDED) – 1970–present, Social Sciences Citation Index (SSCI) – 1970–present, Arts & Humanities Citation Index (A&HCI) – 1975–present, Conference Proceedings Citation Index – Science (CPCI‐S) – 1990–present, Conference Proceedings Citation Index – Social Science & Humanities (CPCI‐SSH) – 1990–present, Emerging Sources Citation Index (ESCI) – 2015–present. **Google (Scholar) search limited to 256 characters and max export of 1000 records. ***Journal of Applied Behaviour Analysis (JABA) searched via www.lens.org with keyword ‘video*’ only.

A total of 6563 records were retrieved from searches, of which 2594 were duplicates. The 3969 unique records were screened by title and abstract and 2376 were excluded. Of the 1593 that were screened at full‐text, 1155 were excluded resulting in a total of 438 studies eligible for inclusion in the current version of the map (Figure 1). Fleiss' kappa demonstrated good agreement overall between raters in the title and abstract screening stage (*κ* = 0.748, 95% CI: 0.720–0.775, *p* < 0.001).

#### 5.1.2 Excluded studies

Of the 3969 records screened, 2376 were excluded at title and abstract screening. A further 1155 were excluded at full‐text screening due to not being available (*n* = 177) or not meeting the EGM's eligibility criteria (*n* = 978). More information on excluded studies can be found at the review's OSF home.

### 5.2 Synthesis of included studies

Whilst 438 studies were included in the current map, the total numbers reported in this section may be higher due to some studies using more than one method and/or intervention. Of the 438 studies included in the map, the majority were published from 2010 onwards (*n* = 354, 80.8%). In terms of study design, most (*n* = 393, 89.7%) were SSRDs and the remainder were RCTs (*n* = 25, 5.7%), non‐randomised group designs (*n* = 14, 3.2%) or pretest–posttest designs (*n* = 8, 1.8%). Not surprisingly, given the number of SSRDs included in the map, sample sizes were primarily under eight (*n* = 361, 87.8%).

#### 5.2.1 Where were studies conducted?

The majority of studies were conducted in the United States of America (*n* = 341, 77.9%). The next largest clusters of evidence came from Turkey (*n* = 18, 4.1%), Australia (*n* = 17, 3.9%), United Kingdom (*n* = 9, 2.1%), New Zealand (*n* = 7, 1.6%) and Canada (*n* = 6, 1.4%).

#### 5.2.2 Study participants

Most studies were carried out in autistic males (*n* = 238, 54.3%); the remainder of studies included both genders (*n* = 172, 39.3%), females only (*n* = 17, 3.9%) or did not report gender of participants (*n* = 11, 2.5%). In terms of age of participants, almost two‐thirds of studies (*n* = 371/616, 60.2%) included autistic children of pre‐school or primary school age (i.e., <12 years) and just over one‐third (*n* = 245/616, 39.8%) included those of secondary school age (i.e., 12+ years). Of note, some studies included multiple age groups and thus appear in both categories reported.

#### 5.2.3 Study interventions

Of the 438 studies in the EGM, 273 (62.3%) evaluated video modelling, 82 (17.7%) video self‐modelling, 61 (13.9%) point‐of‐view modelling, 57 (13.0%) video prompting, 12 (2.7%) video feedback and 4 (0.9%) computer‐based video instruction. In relation to the type of model, 191 studies (43.6%) used adult models, 135 (30.8%) used peers, 96 self‐modelling (21.9%), 26 (5.9%) cartoon/animated models, and 35 (8.0%) studies did not report model type. More than half of studies (*n* = 224/477, 53.5%) conducted their intervention in the school, including pre‐school, setting. Other common settings included the home (*n* = 73/477, 16.7%), clinical (*n* = 56/477, 12.8%) and research (*n* = 46/477, 10.5%) settings.

#### 5.2.4 Types of outcomes

In relation to social outcomes, almost half of studies measured social engagement (*n* = 199/506, 45.4%) and one‐fifth evaluated activities of daily living (*n* = 84/506, 19.2%). Infrequently measured social outcomes included safety (*n* = 9/506, 2.1%) and community (*n* = 7/506, 1.6%) skills.

The current EGM is available to download from the review's OSF page.

## 6 DISCUSSION

### 6.1 Summary of main results

This paper provides a detailed overview of an EGM that summarises the extensive body of evidence on VBIs promoting social behavioural skills for autistic children and young people. The map highlights gaps in the evidence and areas of adequate research in a user‐friendly format that is accessible to a wide range of audiences including autistic people and their families, policy‐makers, practitioners, researchers, and funders. The authors propose that autistic people, their families, and carers can use the map to advocate for services where there is sufficient evidence available. Policy‐makers and practitioners should use the clusters of ample evidence to inform policy and practice on using VBIs to support autistic people. This is of particular importance given the recent policy review that highlighted challenges in identifying specific evidence‐based practices in policy documents across the UK and Northern Ireland (Storey et al., 2023). In addition, researchers and funders should use the map to identify areas for evidence synthesis or direct funding sources to those requiring further research.

The current EGM includes 438 studies, over 300 of which were SSRDs conducted in the USA. The largest cluster of studies evaluated the effectiveness of video modelling on social engagement outcomes. Participants most frequently included in mapped studies were males, two‐thirds of whom were aged under 12 years. Adults were used as models more frequently than peers and studies were most commonly conducted in school settings.

### 6.2 Areas of major gaps in the evidence

This EGM has identified several key gaps in the evidence base related to VBIs promoting social behavioural skills for autistic children and young people.

Of particular interest, very few studies included all female participants compared to over half that included all male participants. Traditionally, autism was considered to be significantly more prevalent in boys compared to girls. However, more recently, research has recognised the diagnostic gender bias towards males (Loomes et al., 2017) and greater camouflaging of autistic traits in females (Wood‐Downie et al., 2021). Given the gender‐based differences in autistic traits (Ratto et al., 2018), research is required to further evaluate VBIs in autistic females.

In spite of the rise in digital technologies, including digital health technology, very few studies used cartoon/animated models, and even fewer evaluated computer‐based video instruction, highlighting the need for continuing evidence building in this area given that interventions of this type continue to be produced. Nonetheless, whilst digital health technologies have much potential for autistic people, they should augment, rather than replace, current practices (Nuske & Mandell, 2021).

Less evidence was conducted in the vocational/work setting, likely reflective of the focus of this EGM on children and young people and the smaller proportion of studies involving older adolescents. Even fewer studies were conducted in community settings despite their value in facilitating research translation and implementation (Wende et al., 2022).

In addition, limited evidence was available for some social outcomes including safety and community skills. Autistic children and young people have an increased risk of injury than their non‐autistic peers (Jain et al., 2014; Lee et al., 2008) and safety skills often need to be explicitly taught to autistic persons from a young age. Therefore, future research should evaluate the effectiveness of VBIs in promoting safety skills for autistic children and young people.

In relation to study design, only 10% of mapped studies employed group designs, and thus research funding should be directed toward sufficiently powered trials.

Finally, the vast majority of mapped studies were conducted in high‐income countries compared to a small proportion (*n* = 29, 6.6%) in low‐ and middle‐income countries. Dependent on the quality of the latter studies, more research may be required in low‐ and middle‐income countries to enhance our social, economic and cultural understanding of autism, particularly in light of immigration from such countries (McConkey, 2022) and the UN Sustainable Development Goals (UN General Assembly, 2015). This may help to inform relevant policies across all countries regardless of their income.

### 6.3 Potential biases in the mapping process

Potential bias was limited in the mapping process by adhering to Campbell Collaboration's guidance for producing an EGM (White et al., 2020). In addition, potential bias was limited by employing a systematic approach and including input from an information retrieval specialist to plan and conduct the searches. Searches also included information sources such as trial registers and repositories to identify the most recent evidence. A further strength was the stakeholder engagement that was carried out during the review process.

### 6.4 Limitations of the EGM

A risk of bias assessment was not conducted due to the large number of studies mapped, although this is not a mandatory process in the development of EGMs. The decision not to include systematic reviews, which should undergo the risk of bias assessment in an EGM, was justified by the comprehensive searches carried out to capture all relevant primary studies in the English language. Risk of bias of primary studies is not required as they will be formally assessed during any subsequent systematic reviews that are carried out by the current review team and other review teams who make use of the map.

### 6.5 Stakeholder engagement throughout the EGM process

Stakeholder engagement for this programme of work is described in Section 4.3. We engaged with stakeholders whilst the review was underway, however we encountered several challenges during the process. Firstly, there is some debate surrounding the need for ethical approval prior to engaging with stakeholders. As our engagement required stakeholders to complete an online survey, we were advised to obtain approval from the School's Research Ethics Committee. Whilst having to seek ethical approval for non‐research activities took time and caused delays in commencing stakeholder engagement, going through this process ensured protection of potentially vulnerable stakeholders. In addition, despite good engagement with the stakeholder engagement survey on Twitter (viewed by >1400 people), only 12 people completed the survey. There was also a lack of people interested in joining an Advisory Group for the review. We propose that our stakeholder engagement activity was less successful than anticipated as we did not engage with stakeholders from the beginning of the review process. Therefore, we will ensure stakeholder engagement is implemented from the start of any further reviews resulting from this EGM.

## 7 AUTHORS' CONCLUSIONS

### 7.1 Implications for research, practice, and/or policy

This paper provides a detailed overview of an EGM that summarises a large body of evidence on VBIs promoting social behavioural skills for autistic children and young people. The map is an accessible resource that identifies areas of sufficient research and highlights important gaps in the available evidence. This information can be used to prioritise research in this field. The EGM included 438 primary studies, of which 90% were single‐subject research designs. The largest volume of research was observed for video modelling and video self‐modelling interventions, with limited evidence available for video feedback and computer‐based video instruction. In addition, the majority of studies evaluated the effectiveness of VBIs on social engagement, activities of daily living, or imaginative play in all‐male samples. A number of factors likely account for the patterns observed in the EGM. For example, the limited number of studies including an all‐female sample reflects the increased prevalence of autism in males compared to females (Loomes et al., 2017).

This EGM's value for researchers, practitioners, and policy‐makers lies in two key interconnected areas; accessibility and allocation of public resources. It has never been more important to ensure that patient and public involvement is a key component of health and social care interventions and this holds the same importance for autistic individuals (Jose et al., 2020). Currently, the mechanisms for autistic people, their families, carers, and associated professionals to access information on evidence‐based practices that may positively impact their lives, rely on their ability to navigate complex policy documents and academic literature (Storey et al., 2023). EGMs remove this barrier to accessing evidence‐based interventions, offering a clear visual representation of where the evidence for a particular intervention is concentrated. For example, if an autistic person wanted to make an evidence‐informed decision about the best way to improve their social engagement skills, they could access the EGM, to identify which VBI has the most supporting evidence for assisting with the development of this skill. It is important that autistic people, their families, carers, and associated professionals are not disadvantaged in accessing evidence if they are not part of academic institutions with free access to journal databases, or if they have difficulty understanding and navigating the complexity of the National Institute for Health and Care Excellence (NICE, 2021) guidelines.

This EGM may positively impact the allocation of public resources. The estimated annual cost of autism in the UK is £3.4 billion, and $66 billion in the United States (Roddy & O'Neill, 2019). Further examination of the distribution of these costs in the UK has uncovered that 95% of this total expenditure relates to state‐provided services (Knapp et al., 2009). Given these figures, it is essential that public resources are allocated to interventions or services where the evidence directly points to their efficacy. The current EGM will allow policymakers to identify evidence‐based approaches which can support autistic people across a range of social behavioural skills, thereby allocating funding for those approaches which are supported by the most evidence.

Similarly, the UK Research and Innovation body receives its money primarily through public taxes, which is redistributed to research organisations and higher education institutions to undertake research and innovation that improves public services generating a prosperous, inclusive, healthy and secure society (UKRI.org). The current EGM has important implications for researchers in the autism field. The map provides an ‘at‐a‐glance’ overview of where particular research areas are saturated (e.g., video‐modelling for teaching daily living skills in all‐male samples) and where further investment should be allocated (e.g., teaching safety skills to autistic females). This ensures that researcher energy is allocated appropriately, to underresearched areas, and that public resources are being allocated to areas of need, as opposed to overresearched areas where further innovation is going to be limited.

In conclusion, this EGM highlights the plethora of research being undertaken to evaluate the effectiveness of VBIs promoting social behavioural skills in autistic children and young people. However, little is known about which VBI is most effective, for whom, and why. Therefore, the second phase of this work, a systematic review and meta‐analysis, is underway to synthesise and analyse this diverse set of interventions to ascertain the usefulness of each VBI and subsequently inform policy and practice. Meta‐analysis on controlled group trials is essential to establish causal relationsips and assess the true efficacy of interventions. Using robust variance estimation, the review team will compare the outcomes of different VBIs, tested using experimental and control groups, to determine which lead to statistically significant improvements in social behavioural skills. In addition to this, the team will investigate which interventions and conditions of the intervention yield the best outcomes for different subgroups of autistic children and young people. This type of analytical approach strenghtens the evidence base and provides reliable insights to interested parties. While the current EGM provides a valuable starting point to this work, controlled trials are essential to answer more nuanced questions about intervention efficacy, refine intervention strategies, and ultimately improve the lives of autistic individuals and their families.

## CONTRIBUTIONS OF AUTHORS

The authors contributed to the EGM as follows.

- Content: all authors
- EGM methods: all authors.
- Statistical analysis: not applicable.
- Information retrieval: Dr. Ciara Keenan and Dr. Karen McConnell.

## DECLARATIONS OF INTEREST

None of the authors have a current or previous affiliation or other involvement with any organisation with an interest in the findings of this EGM that might lead to a perceived or real conflict of interest.

### Plans for updating the EGM

This EGM will be updated every 2 years by the review team as new studies are identified. Any teams interested in building on the EGM or contributing to its updates are encouraged to contact the corresponding author.

## SOURCES OF SUPPORT

### Internal sources

- Support provided by the institutions at which the review was produced, UK.

This EGM has been supported by Queen's University Belfast and Bangor University.

### External sources

- Evidence Synthesis Ireland, Ireland.

Dr. Karen McConnell was part supported by the Health Research Board (Ireland) and the HSC Public Health Agency (Grant number CBES‐2018‐001) through Evidence Synthesis Ireland/Cochrane Ireland.

## DIFFERENCES BETWEEN PROTOCOL AND REVIEW

The published protocol (Keenan et al., 2021) described the planned systematic review and meta‐analysis. However, due to the volume of literature identified during searches, the review team chose to present the evidence visually as an EGM in the first instance. The subsequent systematic review and meta‐analysis are underway and will be prepared for publication in due course. The following planned database searches were removed due to lack of access: Australian Education Index, Canadian Research Index, and Social Science Research Network (SSRN). In addition, FRANCIS (Inist‐CNRS) was removed as adjacency operators did not work, and thus it was not be possible to replicate the search. The review team made the decision to exclude systematic reviews from the map and only include primary studies due to the comprehensive nature of the search and the belief that all relevant primary studies written in the English language were identified. SPSS was used, instead or R statistical software, to calculate Fleiss' kappa to establish overall agreement between raters for all titles and abstracts screened rather than for the first 100 pilot studies. Finally, due to inability to retrieve full text screening comparison data from Eppi‐Reviewer, calculation of inter‐rater agreement was not possible.

## Supporting information

Supporting information.

Supporting information.
