# Supplementary material for: Video‐based interventions promoting social behavioural skills for autistic children and young people: An evidence and gap map
Source: Campbell Syst Rev. 2024 May 3;20(2):e1405. doi: 10.1002/cl2.1405 (PMC11066762; doi:10.1002/cl2.1405)
Supplement: Supplementary file 1 — Supporting information. [file CL2-20-e1405-s001.docx]

**Search Methods**

| **Database (platform)** | **Strategy** | **Search details** | **Added to Eppi?** |
| --- | --- | --- | --- |
| Medline ALL (Ovid) | Electronic database search | Time accessed: 06/05/21 15:53 GMT  Number of hits: 568 | Y |
| PsycINFO® (Ovid) | Electronic database search | Time accessed: 10/05/21 11:15 GMT  Number of hits: 942 | Y |
| Web of Science (Core Collection. Accessed through QUB subscription: 1970 - present) Collection includes:  Science Citation Index Expanded (SCI-EXPANDED) --1970-present  Social Sciences Citation Index (SSCI) --1970-present  Arts & Humanities Citation Index (A&HCI) --1975-present  Conference Proceedings Citation Index- Science (CPCI-S) --1990-present  Conference Proceedings Citation Index- Social Science & Humanities (CPCI-SSH) --1990-present  Emerging Sources Citation Index (ESCI) --2015-present | Electronic database search | Time accessed: 07/05/21 12:25 GMT  Number of hits: 1,179 | Y |
| Education Resources Information Centre (ERIC. EBSCOhost) | Electronic database search | Time accessed: 07/05/21 15:58 GMT  Number of hits: 743 | Y |
| International Bibliography of the Social Sciences (IBSS. ProQuest) | Electronic database search | Time accessed: 13/05/21 13:16 GMT  Number of hits: 19 | Y |
| SCOPUS (ELSEVIER) | Electronic database search | Time accessed: 07/05/21 12:55 GMT  Number of hits: 1,443 | Y |
| British Education Index (BEI. EBSCOhost) | Electronic database search | Time accessed: 07/05/21 16:23 GMT  Number of hits: 40 | Y |
| The Cochrane Central Register of Controlled Trials (CENTRAL) | Electronic database search | Time accessed:  10/05/21 16:30 GMT  Number of hits: 92 (87 trials) | Y |
| FRANCIS (Inist-CNRS) | Free access via <https://pascal-francis.inist.fr/home/> - database stopped in 2015 so records from 1972 to 2015 only - Adjacency operators don’t work – not possible to replicate the search – removed |  |  |
| Australian Education Index | Queen’s no longer subscribe to this |  |  |
| Canadian Research Index | No access to this |  |  |
| Social Science Research Network (SSRN) | No access to this , , |  |  |
| **Grey/ supplementary Literature search** | | |  |
| Google (Scholar) | (autis*\|asperger*\|"pervasive developmental*"\|"triad of impairment*"\|"Fragile X")(video*) (model*\| intervention*\| feedback\| prompt*\|self\|peer*\|"point of view") | Time accessed:  12/05/21 13:46 GMT  Number of hits: 1,000 (limit set by GS) | Y |
| Journal of Applied behaviour analysis (JABA) | Searched the journal of applied behaviour analysis via [www.lens.org](http://www.lens.org) searching for video* only | Time accessed:  12/05/21 13:59 GMT  Number of hits: 149 | Y |
| ProQuest Dissertation and Theses (global) | Electronic database search | Time accessed:  12/05/21 16:35 GMT  Number of hits: 393 | Y |
| Total Records |  | 6562 |  |
| Duplicates Removed |  | 2593 |  |
| Total to screen |  | 3969 |  |

1. **Medline ALL (Ovid)**

| 1 | exp Autistic Disorder/ | 20995 |
| --- | --- | --- |
| 2 | exp Asperger Syndrome/ | 1770 |
| 3 | exp Autism Spectrum Disorder/ | 32380 |
| 4 | (autis* or "pervasive developmental disorder*" or "pervasive developmental delay*" or "pervasive developmental disabilit*" or "global developmental delay*" or asperger* or ASD or HFA or HFASD or " HF-ASD" or SCD or PDD or Rett* or "childhood disintegrative disorder* OR triad of impairment*" or "Fragile X" or PDDNOS or "PDD-NOS" or "PDD/NOS" or savant or "reactive attachment disorder*" or "AS/HFA" or Kanner* or aspies or "childhood schizophrenia" or "atypical personality development*").ti,ab,kw. | 90650 |
| 5 | or/1-4 | 93407 |
| 6 | (video* adj3 (intervention* or feedback or prompt* or model*)).af. | 6956 |
| 7 | (model* adj3 (self or peer* or "in-vivo" or "in vivo")).af. | 54168 |
| 8 | ("Point-of-view" or "Point of view").af. | 44323 |
| 9 | or/6-8 | 105203 |
| 10 | 5 and 9 | 568 |

1. **PsycINFO (1806 - present) (Ovid)**

| 1 | exp Autism Spectrum Disorders/ | 46591 |
| --- | --- | --- |
| 2 | (autis* or "pervasive developmental disorder*" or "pervasive developmental delay*" or "pervasive developmental disabilit*" or "global developmental delay*" or asperger* or ASD or HFA or HFASD or " HF-ASD" or SCD or PDD or Rett* or "childhood disintegrative disorder*OR triad of impairment*" or "Fragile X" or PDDNOS or "PDD-NOS" or "PDD/NOS" or savant or "reactive attachment disorder*" or "AS/HFA" or Kanner* or aspies or "childhood schizophrenia" or "atypical personality development*").ti,ab. | 63730 |
| 3 | or/1-2 | 64796 |
| 4 | (video* adj3 (intervention* or feedback or prompt* or model*)).ti,ab. | 3470 |
| 5 | (model* adj3 (self or peer* or "in-vivo" or "in vivo")).ti,ab. | 13024 |
| 6 | ("Point-of-view" or "Point of view").ti,ab. | 21124 |
| 7 | or/4-6 | 37206 |
| 8 | 3 and 7 | 942 |

1. **Web of Science** (Core Collection. Accessed through QUB subscription: 1970 - present)

QUB subscription includes:

- Science Citation Index Expanded (SCI-EXPANDED) --1970-present
- Social Sciences Citation Index (SSCI) --1970-present
- Arts & Humanities Citation Index (A&HCI) --1975-present
- Conference Proceedings Citation Index- Science (CPCI-S) --1990-present
- Conference Proceedings Citation Index- Social Science & Humanities (CPCI-SSH) --1990-present
- Emerging Sources Citation Index (ESCI) --2015-present

| 1 | TS= autis* OR "pervasive developmental disorder*" OR "pervasive developmental delay*" OR "pervasive developmental disabilit*" OR "global developmental delay*" OR asperger* OR ASD OR HFA OR HFASD OR "HF-ASD" OR SCD OR PDD OR Rett* OR "childhood disintegrative disorder*" OR "triad of impairment*" OR "Fragile X" OR PDDNOS OR "PDD-NOS" OR "PDD/NOS" OR savant OR "reactive attachment disorder*" OR "AS/HFA" OR Kanner* OR aspies OR "childhood schizophrenia" OR "atypical personality development*" | 131,559 |
| --- | --- | --- |
| 2 | TS= video* NEAR/3 (intervention* or feedback or prompt* or model*) | 12,177 |
| 3 | TS= model* NEAR/3 (self or peer* or "in-vivo" or "in vivo") | 98,331 |
| 4 | TS= "Point-of-view" or "Point of view" | 153,675 |
| 5 | #2 OR #3 OR #4 | 263,537 |
| 6 | #1 AND #5 | 1,179 |

Education Resources Information Centre (ERIC. EBSCOhost)

| 1 | DE "Autism" OR DE "Pervasive Developmental Disorders" | 15864 |
| --- | --- | --- |
| 2 | DE "Asperger Syndrome" OR DE "Theory of Mind" | 1953 |
| 3 | TI (autis* or "pervasive developmental disorder*" or "pervasive developmental delay*" or "pervasive developmental disabilit*" or "global developmental delay*" or asperger* or ASD or HFA or HFASD or " HF-ASD" or SCD or PDD or Rett* or "childhood disintegrative disorder*OR triad of impairment*" or "Fragile X" or PDDNOS or "PDD-NOS" or "PDD/NOS" or savant or "reactive attachment disorder*" or "AS/HFA" or Kanner* or aspies or "childhood schizophrenia" or "atypical personality development*") | 13628 |
| 4 | AB (autis* or "pervasive developmental disorder*" or "pervasive developmental delay*" or "pervasive developmental disabilit*" or "global developmental delay*" or asperger* or ASD or HFA or HFASD or " HF-ASD" or SCD or PDD or Rett* or "childhood disintegrative disorder*OR triad of impairment*" or "Fragile X" or PDDNOS or "PDD-NOS" or "PDD/NOS" or savant or "reactive attachment disorder*" or "AS/HFA" or Kanner* or aspies or "childhood schizophrenia" or "atypical personality development*") | 16,649 |
| 5 | KW (autis* or "pervasive developmental disorder*" or "pervasive developmental delay*" or "pervasive developmental disabilit*" or "global developmental delay*" or asperger* or ASD or HFA or HFASD or " HF-ASD" or SCD or PDD or Rett* or "childhood disintegrative disorder*OR triad of impairment*" or "Fragile X" or PDDNOS or "PDD-NOS" or "PDD/NOS" or savant or "reactive attachment disorder*" or "AS/HFA" or Kanner* or aspies or "childhood schizophrenia" or "atypical personality development*") | 551 |
| 6 | or/1-5 | 18256 |
| 7 | DE "Video Technology" | 11,057 |
| 8 | TX video* N3 (intervention* or feedback or prompt* or model*) | 1775 |
| 9 | TX model* N3 (self or peer* or "in-vivo" or "in vivo") | 4,023 |
| 10 | TX ("Point-of-view" or "Point of view") | 5,337 |
| 11 | or/7-10 | 21,057 |
| 11 | 6 AND 11 | 743 |

International Bibliography of the Social Sciences (IBSS. ProQuest)

| 1 | MAINSUBJECT.EXACT("Autism") OR MAINSUBJECT.EXACT("Autistic children") | 1,443 |
| --- | --- | --- |
| 2 | ti(autis* or "pervasive developmental disorder*" or "pervasive developmental delay*" or "pervasive developmental disabilit*" or "global developmental delay*" or asperger* or ASD or HFA or HFASD or " HF-ASD" or SCD or PDD or Rett* or "childhood disintegrative disorder* OR triad of impairment*" or "Fragile X" or PDDNOS or "PDD-NOS" or "PDD/NOS" or "reactive attachment disorder*" or "AS/HFA" or Kanner* or aspies or "childhood schizophrenia" or "atypical personality development*") OR ab(autis* or "pervasive developmental disorder*" or "pervasive developmental delay*" or "pervasive developmental disabilit*" or "global developmental delay*" or asperger* or ASD or HFA or HFASD or " HF-ASD" or SCD or PDD or Rett* or "childhood disintegrative disorder* OR triad of impairment*" or "Fragile X" or PDDNOS or "PDD-NOS" or "PDD/NOS" or "reactive attachment disorder*" or "AS/HFA" or Kanner* or aspies or "childhood schizophrenia" or "atypical personality development*") | 2,630 |
| 3 | or/1-2 | 2,687 |
| 4 | video* NEAR/3 (intervention* or feedback or prompt* or model*) | 230 |
| 5 | model* NEAR/3 (self or peer* or "in-vivo" or "in vivo") | 2,962 |
| 6 | "Point-of-view" or "Point of view" | 14,186 |
| 7 | or/2-4 | 17,328 |
| 8 | 3 and 7 | 19 |

1. SCOPUS (ELSEVIER)

| 1 | TITLE-ABS-KEY (autis* OR "pervasive developmental disorder*" OR "pervasive developmental delay*" OR "pervasive developmental disabilit*" OR "global developmental delay*" OR asperger* OR ASD OR HFA OR HFASD OR "HF-ASD" OR SCD OR PDD OR Rett* OR "childhood disintegrative disorder*" OR "triad of impairment*" OR "Fragile X" OR PDDNOS OR "PDD-NOS" OR "PDD/NOS" OR savant OR "reactive attachment disorder*" OR "AS/HFA" OR Kanner* OR aspies OR "childhood schizophrenia" OR "atypical personality development*") | 154,975 |
| --- | --- | --- |
| 2 | TITLE-ABS-KEY = (video* W/3 (intervention* or feedback or prompt* or model*)) | 16,468 |
| 3 | TITLE-ABS-KEY = (model* W/3 (self or peer* or "in-vivo" or "in vivo")) | 116,974 |
| 4 | TITLE-ABS-KEY = ("Point-of-view" or "Point of view") | 271,928 |
| 5 | #2 OR #3 OR #4 | 404,474 |
| 6 | #1 AND #5 | 1,443 |

Social Science Research Network (SSRN)

British Education Index (BEI. EBSCOhost)

| 1 | DE "AUTISM" OR DE "AUTISM in adolescence" OR DE "AUTISM in children" OR DE "AUTISM spectrum disorders" OR DE "AUTISTIC children" OR DE "AUTISTIC children & education laws" OR DE "AUTISTIC people" | 2,494 |
| --- | --- | --- |
| 2 | DE "EDUCATION of autistic people" OR DE "ASPERGER'S syndrome" OR DE "PERVASIVE developmental disorder not otherwise specified" | 233 |
| 3 | TI (autis* or "pervasive developmental disorder*" or "pervasive developmental delay*" or "pervasive developmental disabilit*" or "global developmental delay*" or asperger* or ASD or HFA or HFASD or " HF-ASD" or SCD or PDD or Rett* or "childhood disintegrative disorder*OR triad of impairment*" or "Fragile X" or PDDNOS or "PDD-NOS" or "PDD/NOS" or savant or "reactive attachment disorder*" or "AS/HFA" or Kanner* or aspies or "childhood schizophrenia" or "atypical personality development*") | 2,526 |
| 4 | AB (autis* or "pervasive developmental disorder*" or "pervasive developmental delay*" or "pervasive developmental disabilit*" or "global developmental delay*" or asperger* or ASD or HFA or HFASD or " HF-ASD" or SCD or PDD or Rett* or "childhood disintegrative disorder*OR triad of impairment*" or "Fragile X" or PDDNOS or "PDD-NOS" or "PDD/NOS" or savant or "reactive attachment disorder*" or "AS/HFA" or Kanner* or aspies or "childhood schizophrenia" or "atypical personality development*") | 1,979 |
| 5 | KW (autis* or "pervasive developmental disorder*" or "pervasive developmental delay*" or "pervasive developmental disabilit*" or "global developmental delay*" or asperger* or ASD or HFA or HFASD or " HF-ASD" or SCD or PDD or Rett* or "childhood disintegrative disorder*OR triad of impairment*" or "Fragile X" or PDDNOS or "PDD-NOS" or "PDD/NOS" or savant or "reactive attachment disorder*" or "AS/HFA" or Kanner* or aspies or "childhood schizophrenia" or "atypical personality development*") | 1,557 |
| 6 | or/1-5 | 3,152 |
| 7 | DE "VIDEO art education" OR DE "VIDEO games" OR DE "VIDEO games in education" OR DE "VIDEO tapes in education" OR DE "VIDEO tapes in sex education" OR DE "VIDEOCONFERENCING in education" OR DE "VIDEODISCS in education" OR DE "VIRTUAL reality in early childhood education" OR DE "VIRTUAL reality in education" OR DE "VIRTUAL reality in higher education" | 387 |
| 8 | TX video* N3 (intervention* or feedback or prompt* or model*) | 209 |
| 9 | TX model* N3 (self or peer* or "in-vivo" or "in vivo") | 308 |
| 10 | TX ("Point-of-view" or "Point of view") | 500 |
| 11 | or/7-10 | 1,387 |
| 11 | 6 AND 11 | 40 |

The Cochrane Central Register of Controlled Trials (CENTRAL)

| 1 | MeSH descriptor: [Autistic Disorder] explode all tress | 1,036 |
| --- | --- | --- |
| 2 | MeSH descriptor: [Asperger Syndrome] explode all tress | 73 |
| 3 | MeSH descriptor: [Autism Spectrum Disorder] explode all tress | 1,537 |
| 4 | (autis* or "pervasive developmental disorder*" or "pervasive developmental delay*" or "pervasive developmental disabilit*" or "global developmental delay*" or asperger* or ASD or HFA or HFASD or " HF-ASD" or SCD or PDD or Rett* or "childhood disintegrative disorder* OR triad of impairment*" or "Fragile X" or PDDNOS or "PDD-NOS" or "PDD/NOS" or savant or "reactive attachment disorder*" or "AS/HFA" or Kanner* or aspies or "childhood schizophrenia" or "atypical personality development*"):ti,ab,kw | 7,269 |
| 5 | #1 or #2 or #3 or #4 | 7,269 |
| 6 | (video* NEAR/3 (intervention* or feedback or prompt* or model*)) | 2,154 |
| 7 | (model* NEAR/3 (self or peer* or "in-vivo" or "in vivo")) | 1,886 |
| 8 | ("Point-of-view" or "Point of view") | 1,362 |
| 9 | #6 or #7 or #8 | 5,357 |
| 10 | #5 and #9 | 87 |

OpenGrey

| 1 | video* NEAR/3 intervention* | 11 |
| --- | --- | --- |
| 2 | video* NEAR/3 feedback | 6 |
| 3 | video* NEAR/3 prompt* | 0 |
| 4 | video* NEAR/3 model* | 47 |
| 5 | model* NEAR/3 self | 301 |
| 6 | model* NEAR/3 peer* | 27 |
| 7 | model* NEAR/3 "in-vivo" | 346 |
| 9 | "Point-of-view" or "Point of view" | 2378 |
| 10 | or/1-9 |  |

Google Scholar

(autis*|asperger*|"pervasive developmental*"|"triad of impairment*"|"Fragile X")(video*) (model*| intervention*| feedback| prompt*|self|peer*|"point of view") **6,140 hits limited to 1000 which is the limit imposed by google.**

ProQuest Dissertation and theses (Global)

| 1 | ti(autis* or "pervasive developmental disorder*" or "pervasive developmental delay*" or "pervasive developmental disabilit*" or "global developmental delay*" or asperger* or ASD or HFA or HFASD or " HF-ASD" or SCD or PDD or Rett* or "childhood disintegrative disorder* OR triad of impairment*" or "Fragile X" or PDDNOS or "PDD-NOS" or "PDD/NOS" or "reactive attachment disorder*" or "AS/HFA" or Kanner* or aspies or "childhood schizophrenia" or "atypical personality development*") OR ab(autis* or "pervasive developmental disorder*" or "pervasive developmental delay*" or "pervasive developmental disabilit*" or "global developmental delay*" or asperger* or ASD or HFA or HFASD or " HF-ASD" or SCD or PDD or Rett* or "childhood disintegrative disorder* OR triad of impairment*" or "Fragile X" or PDDNOS or "PDD-NOS" or "PDD/NOS" or "reactive attachment disorder*" or "AS/HFA" or Kanner* or aspies or "childhood schizophrenia" or "atypical personality development*") | 14,677 |
| --- | --- | --- |
| 2 | video* NEAR/3 (intervention* or feedback or prompt* or model*) |  |
| 3 | model* NEAR/3 (self or peer* or "in-vivo" or "in vivo") |  |
| 4 | "Point-of-view" or "Point of view" |  |
| 5 | or/2-4 |  |
| 6 | 1 and 5 | 393 |
