# Supplementary material for: Video‐based interventions promoting social behavioural skills for autistic children and young people: An evidence and gap map
Source: Campbell Syst Rev. 2024 May 3;20(2):e1405. doi: 10.1002/cl2.1405 (PMC11066762; doi:10.1002/cl2.1405)
Supplement: Supplementary file 2 — Supporting information. [file CL2-20-e1405-s002.docx]

Appendices

Appendix 1. Link to online interactive EGM

https://osf.io/download/r4tzg/
